# Landscape of *in vivo* Fitness-Associated Genes of *Enterobacter cloacae* Complex

**DOI:** 10.3389/fmicb.2020.01609

**Published:** 2020-07-10

**Authors:** François Guérin, Claire Lallement, Benoit Goudergues, Christophe Isnard, Maurizio Sanguinetti, Margherita Cacaci, Riccardo Torelli, Vincent Cattoir, Jean-Christophe Giard

**Affiliations:** ^1^Université de Caen Normandie, EA4655 U2RM (Équipe «Antibio-Résistance»), Caen, France; ^2^CHU de Caen, Service de Microbiologie, Caen, France; ^3^Dipartimento di Scienze Biotecnologiche di Base, Cliniche Intensivologiche e Perioperatorie, Università Cattolica del Sacro Cuore, Rome, Italy; ^4^Dipartimento di Scienze di Laboratorio e Infettivologiche, Fondazione Policlinico Universitario A. Gemelli IRCCS, Rome, Italy; ^5^Institute of Microbiology, Catholic University of the Sacred Heart, Rome, Italy; ^6^Rennes University Hospital, Department of Clinical Microbiology, Rennes, France; ^7^Inserm U1230, University of Rennes 1, Rennes, France

**Keywords:** *E. cloacae*, Tn-seq, fitness, virulence, pathogenicity

## Abstract

Species of the *Enterobacter cloacae* complex (ECC) represent an increasing cause of hospital-acquired infections and commonly exhibit multiple antibiotic resistances. In order to identify genes that may play a role in its ability to colonize the host, we used the transposon-sequencing (Tn-seq) approach. To this end, a high-density random transposon insertion library was obtained from *E. cloacae* subsp. *cloacae* ATCC 13047, which was used to analyze the fitness of ca. 300,000 mutants in *Galleria mellonella* colonization model. Following massively parallel sequencing, we identified 624 genes that seemed essential for the optimal growth and/or the fitness within the host. Moreover, 63 genes where mutations resulted in positive selection were found, while 576 genes potentially involved in the *in vivo* fitness were observed. These findings pointed out the role of some transcriptional regulators, type VI secretion system, and surface-associated proteins in the *in vivo* fitness of *E. cloacae* ATCC 13047. We then selected eight genes based on their high positive or negative fold changes (FCs) and tested the corresponding deletion mutants for their virulence and ability to cope with stresses. Thereby, we showed that ECL_02247 (encoding the NAD-dependent epimerase/dehydratase) and ECL_04444 (coding for a surface antigen-like protein) may correspond to new virulence factors, and that the regulator ECL_00056 was involved in *in vivo* fitness. In addition, bacterial cells lacking the flagellum-specific ATP synthase FliI (ECL_03223) and the hypothetical protein ECL_01421 were affected for mobility and resistance to H_2_O_2_, respectively. All these results yield valuable information regarding genes important for infection process and stress response of *E. cloacae* ATCC 13047 and participate to a better understanding of the opportunistic traits in this bacterial pathogen.

## Introduction

Species of the *Enterobacter cloacae* complex (ECC) are widely encountered in the environment and are also part of the intestinal microbiota of both humans and animals (Sanders and Sanders, 1997). For the last decades, *E. cloacae* has emerged as a major nosocomial pathogen, accounting for up to 5% of bacteremia, 5% of pneumonia, 4% of urinary tract infections, and 10% of postsurgical peritonitis hospital-acquired cases (Fernandez-Baca et al., 2001; Roehrborn et al., 2001). These ubiquitous species are very well adapted to healthcare environments (Chavda et al., 2016), being able to contaminate various medical, intravenous, or other hospital devices while outbreaks usually occur in intensive care units (ICUs), primarily affecting patients with serious comorbidities (Dugleux et al., 1991; Davin-Regli et al., 2019). Moreover, ECC strains usually carry multiple antibiotic genes (Guérin et al., 2015, 2016; Davin-Regli et al., 2019). Little is known about factors impacting the pathogenicity of *E. cloacae*. As for other Gram-negative bacteria, cellular elements such as the type III secretion system (T3SS), T4SS, T6SS, fimbriae, and pili seem to be involved in the development of infection or colonization within the host (Zeng et al., 2003; Bingle et al., 2008; Schwarz et al., 2010; Mulder et al., 2012; Liu et al., 2013; Piqué et al., 2015). In addition, these virulence genes are often associated with pathogenicity islands that may be acquired by horizontal gene transfer (Dobrindt et al., 2004; Juhas et al., 2009). For instance, it has also been shown that resistance and virulence factors in an epidemic *Enterobacter hormaechei* outbreak strain were carried by the conjugative plasmid pQC (Paauw et al., 2009). Currently, clusters belonging to *E. cloacae* (Cluster 3) and *E. hormaechei* (Clusters 6 and 8) are the most frequent clusters of ECC isolated from ICU patients and *Enterobacter bugandensis* (belonging to Cluster 9) appears highly virulent (Dalben et al., 2008; Mezzatesta et al., 2012; Pati et al., 2018).

Recently, several methods based on high-throughput DNA sequencing technologies have been developed for determining the involvement of specific gene products in the steps of bacterial pathogenesis. One of these, referred as Transposon-sequencing (Tn-seq), corresponds to the preparation of a high-density random transposon-insertion library that is then used to compare assessment of all genes to behavior during colonization or under specific environmental conditions (for review, see van Opijnen et al., 2009). Indeed, by sequencing the Tn junctions, it is possible to identify and quantify the role of individual fitness factors because of their over- or under-representation within a pool of mutants. In addition, essential genes crucial for growth in a particular condition (characterized by a lack of corresponding Tn insertions) may be highlighted by this methodology. Such approach has been previously used for a comprehensive analysis of *in vitro* and *in vivo* fitness of numerous Gram-positive and -negative pathogens such as *Pseudomonas aeruginosa*, *Vibrio cholerae*, *Acinetobacter baumannii*, *Klebsiella pneumoniae*, *Borrelia burgdorferi*, *Staphylococcus aureus*, or *Streptococcus pneumoniae* (Maroncle et al., 2002; Kamp et al., 2013; Skurnik et al., 2013; Lin et al., 2014; Valentino et al., 2014; Verhagen et al., 2014; Gallagher et al., 2015).

Tn-seq appears then as an interesting tool to assess the genetic traits that play a role in the ability of *E. cloacae* ATCC 13047 to switch from a commensal state to an opportunistic pathogen. In this study, a *Galleria mellonella* colonization model was used in conjunction with transposon mutagenesis to identify bacterial mechanisms involved in the host survival establishing. Transposon mutants that survived into the larvae were isolated and insertion sites in the input and output samples were mapped to the genome of *E. cloacae* subsp. *cloacae* ATCC 13047. Putative essential genes as well as novel fitness and virulence factors were thus identified. Among them, we selected eight genes whose knockout mutants were constructed. Phenotypic studies of these last strains allowed us to characterize previously unknown fitness and virulence factors in *E. cloacae* ATCC 13047.

## Materials and Methods

### Strains, Media, and Growth Conditions

Bacterial strains and plasmids used in this study are listed in Table 1. The reference strain used in the study was *E. cloacae* subp. *cloacae* ATCC 13047 (belonging to the cluster XI). This strain was isolated from human cerebrospinal fluid and corresponds to the type strain of *E. cloacae* subsp. *cloacae* (Ren et al., 2010). It was also the first reference strain fully sequenced and annotated (GenBank accession numbers CP002886, FP929040, and AGSY00000000) (Ren et al., 2010). *Escherichia coli* and *E. cloacae* strains were cultured with shaking (200 r/min) at 37°C in Luria-Bertani (LB) medium with streptomycin (80 μg/ml), vancomycin (100 μg/ml), chloramphenicol (25 μg/ml), or gentamicin (15 μg/ml) when required.

**TABLE 1:** Bacterial strains and plasmids used in this study.

**Strains/plasmids**	**Characteristics^a^**	**Origin**
**Strains**		
ATCC 13047	*E. cloacae* subp. *cloacae* ATCC 13047	Ren et al., 2010
*E. coli* SM100 λ*pir*	*E. coli* strain with λ*pir* (encoding R6K trans-acting protein)	Miller and Mekalanos, 1988
ECLΔ00056KR	Deletion of ECL_00056 gene of ATCC 13047/Kan^r^	This study
ECLΔ00056	Deletion of ECL_00056 gene of ATCC 13047/Kan^s^	This study
ECLΔ00095KR	Deletion of ECL_00095 gene of ATCC 13047/Kan^r^	This study
ECLΔ00095	Deletion of ECL_00095 gene of ATCC 13047/Kan^s^	This study
ECLΔ00417KR	Deletion of ECL_00417 gene of ATCC 13047/Kan^r^	This study
ECLΔ00417	Deletion of ECL_00417 gene of ATCC 13047/Kan^s^	This study
ECLΔ01421KR	Deletion of ECL_01421 gene of ATCC 13047/Kan^r^	This study
ECLΔ01421	Deletion of ECL_01421 gene of ATCC 13047/Kan^s^	This study
ECLΔ02046KR	Deletion of ECL_02046 gene of ATCC 13047/Kan^r^	This study
ECLΔ02046	Deletion of ECL_02046 gene of ATCC 13047/Kan^s^	This study
ECLΔ02247KR	Deletion of ECL_02247 gene of ATCC 13047/Kan^r^	This study
ECLΔ02247	Deletion of ECL_02247 gene of ATCC 13047/Kan^s^	This study
ECLΔ03223KR	Deletion of ECL_03223 gene of ATCC 13047/Kan^r^	This study
ECLΔ03223	Deletion of ECL_03223 gene of ATCC 13047/Kan^s^	This study
ECLΔ04444KR	Deletion of ECL_04444 gene of ATCC 13047/Kan^r^	This study
ECLΔ04444	Deletion of ECL_04444 gene of ATCC 13047/Kan^s^	This study
**Plasmids**		
pKD4	Plasmid containing an FRT-flanked kanamycin cassette, Kan^r^	Datsenko and Wanner, 2000
pCP20	Ampicillin and Cm^r^ plasmid that shows temperature-sensitive replication and thermal induction of FLP synthesis	Cherepanov and Wackernagel, 1995
pKOBEG	Recombination vector, phage λ recγβα operon under the control of the pBAD promoter, Cm^r^	Derbise et al., 2003
pSAM_DGm	Amp^r^ Gen^r^; suicide delivery vector with mariner transposase himar1c9	Skurnik et al., 2013

*^*a*^Amp^*r*^, ampicillin resistant; Cm^*r*^, chloramphenicol resistant; Gen^*r*^, gentamicin resistant; Kan^*r*^, kanamycin resistant; Kan^*s*^, kanamycin susceptible.*

### Transposon Library Construction

The pSAM_DGm vector carried by *E. coli* SM10λ*pir* was delivered to ECL13047 by mating (Miller and Mekalanos, 1988; Hoffmann and Roggenkamp, 2003; Skurnik et al., 2013). The donor strain *E. coli* SM10λ*pir* was grown overnight in LB with 10 μg/ml gentamicin and the recipient *E. cloacae* ECL13047 in LB without antibiotics. Cells were centrifuged, washed in LB, and re-suspended to an OD_600_ of 2.0. Equal volumes of both strains were mixed and 100 μl of the suspension was spread onto pre-warmed LB agar plates. For the preparation of the library, 100 independent conjugation mixtures were performed. The plates were left at room temperature for 10 min and the mating continued for 3 h at 37°C. Each conjugation reaction was scraped off, suspended in 10 ml LB and 150 μl were plated on LB plates containing 15 μg/ml gentamicin and 80 μg/ml streptomycin. After 14 h at 37°C, the plates were flooded with LB and the colonies were scraped off and pooled. The yield was approximately of 300,000 clones from 100 plates. The library was further incubated for 2 additional hours in LB broth with gentamicin. Following centrifugation, the high-density transposon library was re-suspended in LB containing 20% glycerol and aliquots were frozen at –80°C.

### *Galleria mellonella* Model of Colonization, Virulence, and Competition Experiments

A volume of 250 μl of the library was re-suspended in 100 ml LB containing 15 μg/ml gentamicin and incubated overnight at 37°C. Cells were centrifuged, washed in saline buffer (0.9% NaCl), and re-suspended to an OD_600_ of 0.1 (3 ± 0.6 10^7^ CFU/ml). Non-lethal bacterial inoculums (10 μl corresponding to 3 × 10^5^ CFU/larva) were injected dorsalaterally into the hemocoel of larvae using a microinjector (KDS100, KD Scientific, Holliston, MA, United States). Groups of 10 alive insects were collected 48 h post-infection and were homogenized in 9 ml of saline buffer (0.9% NaCl) using UltaTurrax breaker (IKA, Staufen, Germany). One ml of each mixture was re-suspended in 20 ml LB containing 15 μg/ml gentamicin and 100 μg/ml vancomycin in order to kill *Enterococcus casseliflavus* cells present in the normal flora of the larvae and incubated at 37°C overnight.

The virulence of *E. cloacae* ATCC 13047 and derived strains was tested in the *G. mellonella* model of infection. To this end a suspension of OD_600 nm_ of 0.5 (4.0 ± 1.0 10^8^ CFU/ml) were injected dorsolaterally into the hemocoel. After injection, the larvae were incubated at 37°C and survival of the larvae was evaluated until 72 h post-infection. Competition experiments were performed with each of the isogenic pairs: ECL13047/ECLΔ00056KR, ECL13047/ECLΔ00095KR, ECL130 47/ECLΔ00417KR, ECL13047/ECLΔ01421KR, ECL13047/ECL Δ02046KR, ECL13047/ECLΔ02247KR, ECL13047/ECLΔ03223 KR, and ECL13047/ECLΔ04444KR. Exponentially growing cells in LB broth were harvested, washed, and resuspended in saline buffer (0.9% NaCl) in order to obtain an OD_600 nm_ of 0.1. A mixture 1:1 of each isogenic pairs was realized. Infection of *G. mellonella* larvae and bacterial cells recovery were carried out as describe above. Serial 10-fold dilutions were plated on LB agar and on LB agar containing 40 μg/ml of kanamycin, to determine the total CFU and the CFU of the mutants, respectively. The competition index (CI) was defined as the ratio of mutant/wild-type numerations. The CI values were calculated for each of the eight independent competition experiments performed in triplicate, and the mean values were recorded.

### DNA Preparation and High-Throughput Sequencing

The protocol used was as previously described by Skurnik et al. (2013). Briefly, total DNA from the bank of transconjugants recovered after 24 h of growth in LB media or after 48 h inside *G. mellonella* (see above) was extracted and purified using the Nucleobond Xtra Plus kit (Macherey-Nagel, Düren, Germany). DNA samples were then digested with *Mne*I restriction enzyme that cuts 20 bp away from the recognition site present in the transposon (thus 16 bp outside of the Tn insertion). After gel extraction of the fragments carrying the transposon and adjacent genomic DNA (1.2–1.5 kb) (Gel Extraction Kit, Qiagen), the adaptors were ligated. PCR amplification of both ends of these fragments was carried out using specific primers: LIB_PCR_5’ complementary to the P7 Illumina sequence present in the transposon, and LIB_PCR_3’ which anneals to the adaptor sequence (Supplementary Table S1). The 125 bp-PCR products were gel extracted, quantified by Biospec-nano (Shimadzu, Marne la Vallée, France) analysis, and sequenced using an Illumina Hiseq 2500 benchtop sequencer (ProfileXpert-LCMT, Lyon, France). Sequence analysis was performed using the CLC Genomic WorkBench software (Qiagen, Valencia, CA, United States) by comparison with the annotated sequence of the *E. cloacae* ATCC 13047 strain. From the Illumina sequencing data, a total of 7,554,249 (from LB sample) and 8,995,232 (from the T48h sample) reads were obtained. Of these, 1,785,967 (from LB) and 1,269,660 (from T48h) mapped uniquely to the genome of *E. cloacae* ATCC 13047 (Table 2).

**TABLE 2:** Summary of Tn-seq coverage data.

**Statistics**	**LB**	**T48**
Total number of reads	7,554,249	8,995,232
Number of uniquely mapped reads	1,785,967	1,269,660
Percentage of uniquely mapped reads (%)	23.64%	14.11%
Number of genes^b^ with no reads	435	320
Mean of reads by gene	371	258

*^*a*^The genome size of ECL13047 is 5,314,588 bp. ^*b*^The number of predicted genes is 5241.*

The sequences of the transposon–genome junction reads were mapped on the *E. cloacae* ATCC 13047 genome. After removal of adapter and transposon sequences, number of reads for each open reading frames were normalized by calculating the “reads per kilobase per million reads” (RPKM) using the CLC Genomic WorkBench software (Qiagen). The fold change (FC) for one specific gene corresponded to the ratio of RPKM from the “T48” sample (48 h into the larvae) to the RPKM from the “LB” sample (24 h growth in LB media). Statistical analysis of the RPKM between the samples was carried out using the CLC software and genes identified as involved in the survival within larvae were selected when the difference in abundance of the transposon mutant was >10 or <10 and with *P*-value < 0.05. In order to avoid the reads corresponding to over-representative mutants present into the library, the 17 genes with RPKM values higher than the mean plus twofold standard deviation were not included in the analysis. Data are available in the NCBI database, BioProject accession number PRJNA627053.

### Construction of the Knockout Mutants

Based on TnSeq data, deletion of selected genes putatively involved in the *in vivo* fitness was performed using the double crossing-over method previously described with some modifications, using the plasmid pKOBEG (Cherepanov and Wackernagel, 1995; Datsenko and Wanner, 2000; Derbise et al., 2003). This last is a low copy number vector that contains a gene for chloramphenicol resistance selection, a temperature-sensitive origin of replication and a gene encoding a recombinase. Briefly, pKOBEG was first introduced into the competent cells of *E. cloacae* ATCC 13047 by electroporation, and transformants were selected on LB agar with chloramphenicol (25 μg/ml) after incubation for 24 h at 30°C. A selectable kanamycin resistance cassette [flanked by Flippase Recognition Target (FRT) sequences] was amplified by PCR using DNA of pKD4 plasmid as template. The primers used included 5’ extensions with homology for the candidate genes (around 50 bases) (Supplementary Table S1). The PCR product was introduced in the *E. cloacae* ATCC 13047/pKOBEG by electroporation and after homologous recombination the disruption of candidate gene was obtained. Selected clones (resistant to chloramphenicol and kanamycin) were cured for the pKOBEG plasmid following a heat shock creating the kanamycin resistant and chloramphenicol susceptible strains (Table 1). In order to have deletion mutants without the antibiotic marker, strains were then transformed with the pCP20 plasmid able to express the FLP nuclease that recognize the FRT sequences present on either sides of the *kan* gene. Lastly, the mutants were verified by sequencing regions surrounding the deletion.

### Phenotypic Analysis of Deleted Mutants

MICs of 23 different antibiotics, four antiseptics, five biocides, five heavy metal ions (listed in Supplementary Table S2), and SDS were determined by the microdilution method in Mueller–Hinton broth in three independent experiments, as previously described (Guérin et al., 2014).

The whole-cell autolysis assays were carried out as following: overnight cultures were diluted to an OD_600_ of 0.1 in 24 ml LB in a 125-ml flask. Cultures were incubated at 37°C with shaking at 200 r/min. After 4.5 h, the cultures were centrifuged for 10 min at 4100 r/min, washed twice with 12.5 ml cold water, and re-suspended in autolysis buffer [50 mM Tris-HCl (pH 7.2) with 0.5% Triton X-100]. At 30 min intervals, samples were shacked and the OD_600_ was measured using spectrophotometer. Data are reported as the percent of the initial OD_600_ for each sample.

To test the bacterial mobility, swimming assays were done by spotting 5 μl of a log-phase culture on 0.5% LB agar plates. The swimming zone was measured after overnight incubation at 37°C.

For the H_2_O_2_ and acid pH challenges, wild-type and mutant cells (log-phase culture) were harvested at an OD_600 nm_ of 0.1 by centrifugation and resuspended in distilled water with 20 mM H_2_O_2_ or LB adjusted to pH 5, respectively. These cultures were incubated at 37°C during 30 min. Before and after the challenge, samples were taken for plate count. The number of CFU was determined after 24 h incubation at 37°C. Each value was the mean of at least three experiments and statistical comparison of means was performed by using Student’s test. Survival was determined as the ratio of the number of CFU after treatment to the number of CFU at the zero time point.

## Results

### Generation of a High-Density Transposon Library of *E. cloacae* and Identification of Genes Contributing to Growth and *in vivo* Fitness

We constructed a library of ca. 300,000 mutants by Tn insertions in the *E. cloacae* ATCC 13047 strain, which was then used to colonize larvae of *G. mellonella* in order to identify fitness-associated genes.

We performed comparative analysis of Tn-seq data obtained from bacterial cells harvested after 24 h of growth in LB (LB condition) and after 48 h into the host (T48 condition). Analysis of the Tn-insertion positions showed an even distribution throughout the chromosome with a statistical average spacing between Tn insertions of 19 bp. Because the Tn-seq experiments were performed with a high density pool of mutants grown in competition, it was difficult to distinguish slow-growing and non-growing cells. Among the 5241 genes annotated in the *E. cloacae* ATCC 13047 chromosome, 624 (11.9%) were found to lack transposon insertion (or with less than 1 insertion/kb of coding sequence) in at least one of the two conditions tested (Supplementary Tables S3, S4). Interestingly, 435 genes were referenced as “necessary for growth in LB” (no read in LB growth condition) (Supplementary Table S3) while 189 as “contributing to *in vivo* fitness” (reads in LB growth condition, but none in the T48 condition) (Supplementary Table S4). Analysis of the functional categories revealed that 295 of them (47%) corresponded to genes coding for hypothetical proteins, 81 for tRNAs, and 47 for ribosomal proteins, rRNA or translation initiation factors (Supplementary Tables S3, S4). Moreover, the other genes classified as essential belonged to the diverse functional categories such as regulation, transport, metabolism, or stress response (Supplementary Tables S3, S4). Among these genes, the putative virulence factor *impE* (coding for the T6SS protein ImpE) was found as necessary for the survival of *E. cloacae* ATCC 13047 into the host (Supplementary Table S4).

### Identification of Positively Selected Tn-Insertion Mutants

Genes with Tn insertions leading to enhanced survival within *G. mellonella* (corresponding to positively selected mutants) were selected based on a ≥10-fold increase in sequencing reads of the output bacterial population within the host (T48h) compared to the LB input corresponding to a “strong selection” (Gallagher et al., 2011). Only 63 genes where mutations resulted in positive selection during the larvae infection were retrieved (Supplementary Tables S5, S6). Proteins coded by the four genes showing the highest FCs (from 106- to 124-fold) were the putative catalase (ECL_02046) and three surface-associated proteins [pilin accessory protein PilO (ECL_00417), surface antigens-like protein (ECL_04444), and sodium:citrate symporter family protein (ECL_01883)] (Supplementary Table S6). In addition, 12 other gene products linked to the cell membrane (transporter, membrane proteins, and flagella) were positively selected (corresponding mutants more retrieved) during the colonization of the insect larvae. Whereas numerous mutants affected in genes involved in the structure or synthesis of flagella were negatively selected (see below), Tn insertion in *fliM* (coding for the flagellar motor switch protein FliM) and ECL_01419 (encoding the flagellar biosynthesis protein FlhA) led to enhanced development in larvae (Supplementary Table S6). Beside loci coding for hypothetical proteins and metabolic enzymes, several cells with Tn insertion in genes encoding regulators were also over-represented after 2 days spent inside the caterpillar. Therefore, two putative regulatory proteins (ECL_00272, ECL_00095), two LysR-type transcriptional regulators (ECL_03990, ECL_04180), one two-component system of the NtrC family (ECL_03891, ECL_03893), one NifA subfamily regulator, and the SgrR regulator (ECL_00865) were identified (Supplementary Table S6). In *Enterobacteriaceae*, SgrR coordinates the cytoplasmic accumulation of phosphorylated sugars mainly by interacting with SgrS, a small regulatory RNA that has an important role on metabolism, growth physiology, and pathogenesis (Vanderpool and Gottesman, 2007; Bobrovskyy and Vanderpool, 2014).

### Negative Selection of Tn Insertion Mutants During Infection of *G. mellonella*

As expected, much more negatively selected Tn-insertion mutants (less retrieved *in vivo*) than positively selected were obtained. Our screen allowed us to identify 576 genes potentially involved in the *in vivo* fitness, as the corresponding mutants from the library were significantly under-represented after 48 h into the host compared to the pool grown in LB (FC < 10 fold) (Supplementary Tables S5, S7). Except the 141 genes (24%) that were annotated as hypothetical, most of the others seemed involved in transport and cellular metabolism.

One interesting feature among the list of genes that may contribute to the *in vivo* fitness was the important proportion of putative or known transcriptional regulators and regulatory proteins (44 genes). This highlights the very subtle regulatory networks that take place when the cells have to face the host environment. Moreover, we identified clusters (20 genes) of Tn-insertion in genes annotated as flagellar proteins or involved in the flagella biosynthesis. They corresponded to the putative operons *flg*_1 (with the genes B, C, D, E, F, G, H, J, and L) and *fli*_1 (genes E, F, G, I, L, N, O, P, and R), *fliH*_2, and *flhA* (Supplementary Table S8). Note that four of the 14 candidates showing an FC lower than 100-fold (from -105 to -677) were associated to the bacterial flagella. In addition, as shown in Supplementary Tables S5, S8, five genes encoding proteins related to the presence of this motility organelle were classified as necessary for the *in vivo* fitness into the host. Taken altogether, flagella appeared as a key factor involved in the host colonization by *E. cloacae*.

### Phenotypic Validation of Fitness-Associated Genes Candidates

In order to validate some candidates identified by the Tn-seq screen, we picked up eight genes among the positively and negatively selected mutants (more and less retrieved *in vivo*, respectively) showing the most obvious FCs and with putative function likely linked to the opportunistic feature of *E. cloacae*, (i.e., genes coding for a regulator or proteins potentially involved in motility or in stress response). The list and genetic organizations of the selected candidates are presented in Table 3 and Supplementary Figure S1. Then a large panel of phenotypic tests were performed. It included mobility, induction of autolysis, MICs to antibiotics and biocides whose are factors of selective pressures for the emergence of *E. cloacae* strains especially in hospital environment. Likewise, we tested the abilities to cope with oxidative and acid pH stresses that are important to escape the host defenses during the immune response. In addition, each mutant has been evaluated for its virulence and its ability of co-colonize larvae with the wild-type strain.

**TABLE 3:** Genes selected for specific deletion.

**Gene**	**Gene product**	**Fold change**	**Involvement**
ECL_00056	TetR family transcriptional regulator	–169	Gene important for *in vivo* fitness
ECL_00095	Putative transcriptional regulatory protein	+75	Gene deleterious for *in vivo* fitness
ECL_00417	Pilin accessory protein PilO	+121	Gene deleterious for *in vivo* fitness
ECL_01421	Hypothetical protein	–677	Gene important for *in vivo* fitness
ECL_02046	Putative catalase	+124	Gene deleterious for *in vivo* fitness
ECL_02247	NAD-dependent epimerase/dehydratase family protein	–304	Gene important for *in vivo* fitness
ECL_03223	Flagellum-specific ATP synthase = FliI_1	–211	Gene important for *in vivo* fitness
ECL_04444	Opacity protein and surface antigens-like protein	+107	Gene deleterious for *in vivo* fitness

No significant difference was shown for any of the mutants on the optimal standard growth in LB at 37°C (Supplementary Figure S2). In addition, susceptibilities to antibiotics, antiseptics, biocides, and heavy metals were not significantly different between mutant and wild-type strains (Supplementary Table S2). No dissimilarity was either observed for the response to acid stress (pH = 5) or bacterial survival after autolysis induction triggered by addition of 0.5% Triton X100 (Supplementary Figure S3).

**FIGURE 1:**
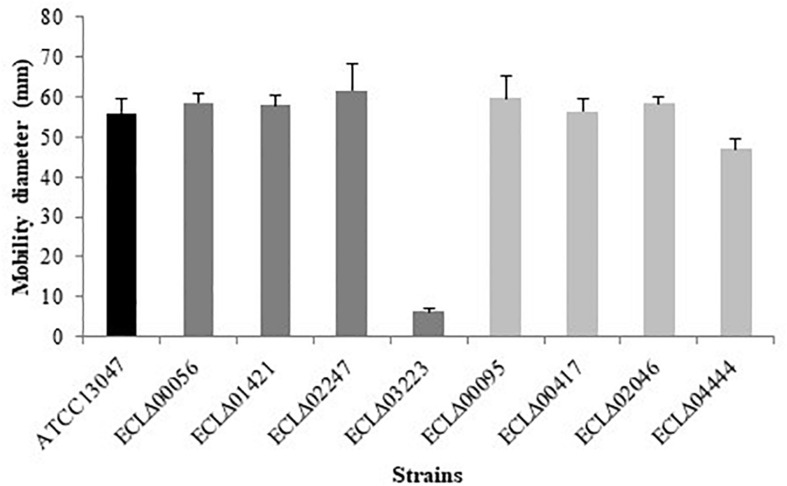
Motility assay on 0.5% agar TS medium. Diameter of *E. cloacae* ATCC 13047 and mutants strains.

Each strain was seeded on a TS semi-solid agar (0.5% agar) in order to observe their ability to move. As expected, only the ECLΔ03223 deletion mutant (gene encoding the flagellum-specific ATP synthase FliI) showed a lack of mobility compared to the wild-type strain (Figure 1).

**FIGURE 2:**
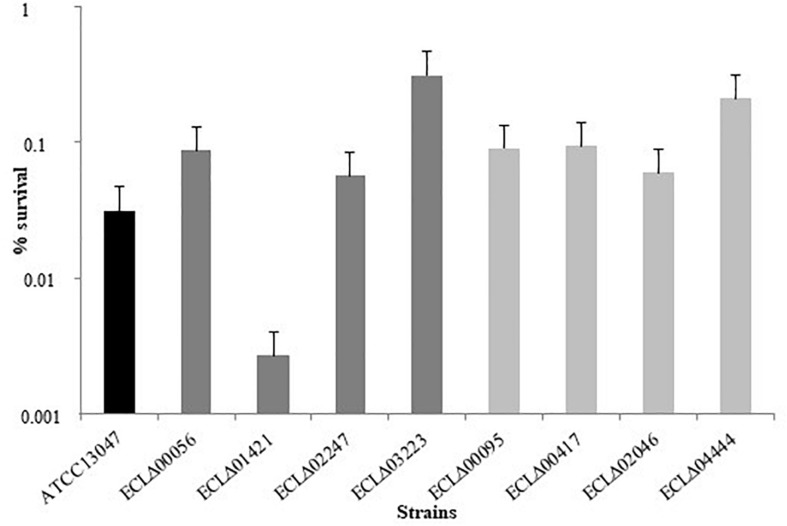
Percentage of survival of the different *E. cloacae* strains after 30 min in the presence of 20 mM H_2_O_2_.

To evaluate their ability to withstand oxidative stress, mutants were then subjected to a hydrogen peroxide challenge (20 mM). As shown in Figure 2, only the ECLΔ01421 mutant (gene encoding a hypothetical protein) was more susceptible (0.0018% of survival) than the wild-type strain (0.03% of survival) after 30 min in the presence of H_2_O_2_ (*p* = 0.01). Surprisingly, no phenotype was observed for the ECLΔ02046 mutant strain devoid of the putative catalase that was suspected to detoxify cells from hydrogen peroxide molecules.

**FIGURE 3:**
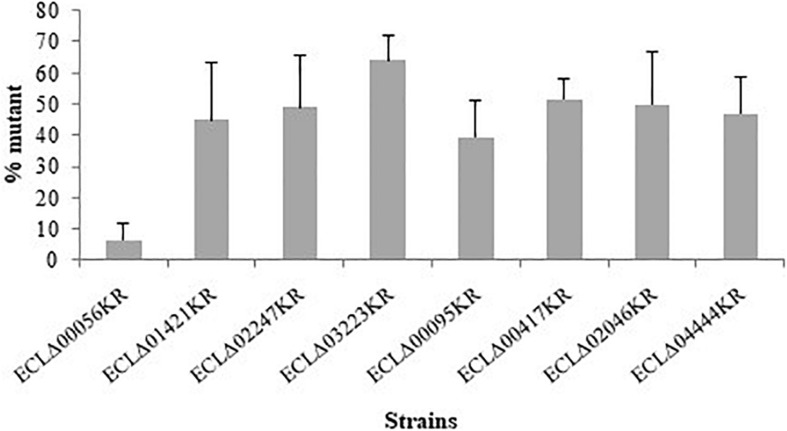
Competition assays of the different mutant strains (kanamycin resistant) competed with the wild-type *E. cloacae* ATCC 13047 when both strains were inoculated together (50/50%) (around 3 × 10^5^ CFU/larvae). Bars represent the percentage of the mutant cells numbered after 48 h into the larvae of *G. mellonella*. Results are the average of, at least, three independent experiments and the represent the mean ± standard deviation.

*In vivo* co-colonization experiments with the wild-type strain and each deleted mutants (ratio 1:1) were performed and the ratios between the two types of bacterial cells were determined 48 h post-injection in *G. mellonella*. In order to discriminate the wild-type and mutant strains, plate counts were carried out with and without kanamycin since mutants were resistant to this antibiotic. We observed that the strain deleted for ECL_00056 gene (coding for a TetR family transcriptional regulator) was present in lower proportion compared to the wild type (6.5% of remaining mutants) after 48 h of incubation into the larvae of *G. mellonella* (Figure 3). Analysis of the chromosomal region surrounding ECL_00056 suggested that it may be the first gene (and the regulator) of the *eefABC* operon encoding a multi-drug efflux transporter. Transcriptomic study of *eefA* by qRT-PCR revealed that this gene was more than 70-fold transcribed in the ECLΔ00056 mutant compared to the *E. cloacae* ATCC 13047 parental strain (data not shown).

**FIGURE 4:**
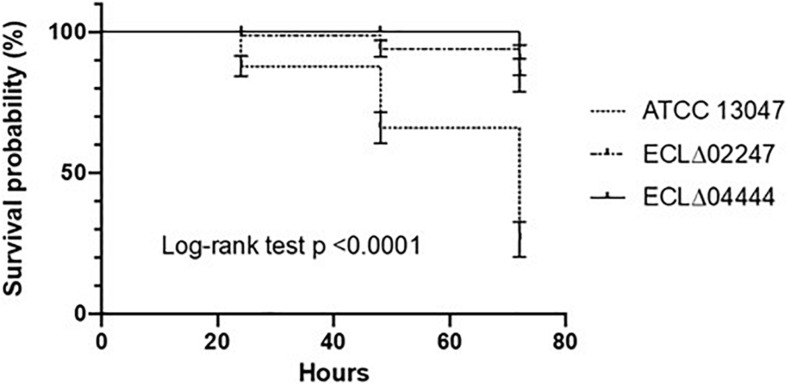
Effect of gene deletions on virulence. Percent survival of *G. mellonella* larvae over 72 h after infection with around 6 × 10^6^ CFU of *E. cloacae* ATCC 13047, ΔECL_02247 mutant, and ECLΔ04444 mutant strain. Experiments were repeated at least three times, and the results represent the mean ± standard deviation of live larvae.

Lastly, the different mutant strains were inoculated (ca. 6.10^6^ cells) into the larvae of *G. mellonella* in order to test their virulence. The number of surviving larvae was observed after 24, 48, and 72 h of incubation at 37°C. As shown in Figure 4, the wild-type strain killed nearly half of the insects at 48 h post-infection and almost two-thirds died after 72 h (Figure 4). Strain deleted for the ECL_02247 gene (encoding a NAD-dependent epimerase/dehydratase family protein), as well as for the ECL_04444 gene (annotated as the opacity protein and surface antigens-like protein) were less virulent: when infected by one of these mutants, 90% of the larvae were still alive at 72 h post-infection (Figure 4) (*p*-ECL_02247 = 0.01; *p*-ECL_04444 = 0.04).

## Discussion

We used the Tn-seq approach to globally identify genes contributing to *E. cloacae* fitness in the *G. mellonella* model of infection. It is a well-established model to study virulence factor in numerous bacteria including *E. cloacae* ATCC 13047 (Guérin et al., 2016). The *G. mellonella* immune response has structural and functional similarities with the innate immune response of mammals and this insect has been extensively used as an efficient infection model to evaluate the virulence of numerous obligate and opportunistic pathogens (Kavanagh and Reeves, 2004; Gaspar et al., 2009; Lebreton et al., 2009). In addition, these larvae can be incubated at 37°C and surviving bacterial cells may be easily recovered (Lebreton et al., 2011).

To our knowledge, this is the first time that this technique has been used for *Enterobacter* while the Tn-insertion mutant library we generated is an interesting tool for further studies with the aim to characterize genes involved in a particular environment (i.e., presence of antibiotics, stress response, other infection models). Of the 624 genes necessary for the growth in rich media or the survival within the host (no transposon insertion found), 295 coded for proteins of unknown function. Their study using for example global approaches (genomics, transcriptomics, or proteomics) may constitute a field of investigation to find new putative targets of anti-bacterial strategies (Kamp et al., 2013; Skurnik et al., 2013; Valentino et al., 2014; Verhagen et al., 2014; Gallagher et al., 2015). As pointed out for *S. aureus*, most of these essential regions were less than 200-bp long suggesting that some may correspond to genes encoding small regulatory RNAs with a crucial role in the control of expression of nearby genes involved in the fitness (Valentino et al., 2014).

The T6SS is a mechanism for protein transport across the cell envelope of Gram-negative bacteria and has been shown to be a key virulence factor for some pathogens because of its implication in the translocation of a potential effector into eukaryotic cells (Bingle et al., 2008; Schwarz et al., 2010; Mulder et al., 2012). However, it appears that the main role of T6SS is the injection of toxins into other neighboring bacteria after cell–cell contact. These secretion systems play an important role in interbacterial competition and the T6SS-mediated antibacterial activity of enteric microoganisms influence the intestinal microbiota and host health (Sana et al., 2017). It has been proposed that *E. cloacae* strains (including ATCC 13047) possessing more than one T6SS cluster may have fitness advantages in a broader range of environments (Liu et al., 2013). In addition, the prevalence of T6SS in pathogenic *E. coli* strains strongly suggests that these secretion systems may have important functions in virulence (Navarro-Garcia et al., 2019). In this context, it was not surprising to find *impE* (encoding the type VI secretion system protein ImpE) as essential for the survival in the host. It is also interesting to note that few reads have been found for the two T6SS gene clusters (*vasD-E*, *impKLMABC* and *impE-H*, *vasG*) (Supplementary Table S5). These data are in favor of the perspective to T6SS for potential therapies (Costa et al., 2015).

Among genes with critical roles in the *in vivo* fitness, we identified many loci encoding flagellar proteins or involved in their biosynthesis. It has been evidenced that motility and chemotaxis are important factors in enterobacterial pathogenicity. For instance, flagella and toxins promote dissemination of uropathogenic *E. coli* and enable *Salmonella* species to travel to the epithelial barrier after ingestion (de Jong et al., 2012; Lüthje and Brauner, 2014; Piqué et al., 2015). Moreover, flagellin from *Salmonella* causes upregulation of pro-inflammatory cytokines in tissue culture models (Zeng et al., 2003). In *E. cloacae* ATCC 13047, we showed, on the one hand, that the ECL_03223 gene (encoding the flagellum-specific ATP synthase FliI) was one of the negatively selected candidate with the lowest FC (-211), and, on the other hand, that the corresponding deletion mutants had lost its motility. These are strong arguments to consider flagella and motility as major *in vivo* associated-factors in *E. cloacae* that participate to its pathogenicity.

In this work, we attempted to characterize potentially interesting gene candidates selected based on their important FC in our Tn seq analysis and their putative function. We performed phenotypic studies of mutants for four genes where Tn insertion resulted in positive selection during colonization of the larvae of *G. mellonella*. Until yet, no phenotype was observed for the mutants deleted for ECL_00095 (coding a putative transcriptional regulatory protein with a DNA binding site), ECL_00417 (coding the pilin accessory protein PilO), and ECL_02046 (coding a putative catalase). The high positive FC demonstrated for the strain with Tn insertion in the gene coding this putative catalase [homologous to the manganese (Mn)-dependent catalase in several enterobacterial species] revealed an obvious selective advantage of this mutant within the Tn library. It has already been observed that a strain of *Lactobacillus plantarum*, which was unable to produce manganese (Mn) catalase, grew more rapidly and to a slightly greater density than did a (Mn) catalase-positive strain (Kono and Fridovich, 1983). Moreover, the absence of phenotype in the oxidative stress response for a (Mn) catalase deficient mutant of *E. cloacae* is consistent with the weak contribution of such enzyme to the overall catalase activity observed in *Salmonella* and suggests that the other catalases have a compensatory effect (Robbe-Saule et al., 2001).

The enhanced colonization by the strains defective in the synthesis of the PilO protein (ECL_00417) and the polypeptide annotated as opacity protein and surface antigens-like protein (porin family) (ECL_04444) may mean that these likely surface-exposed proteins correspond to targets recognized by the host defense mechanisms. Positive selection of Tn-insertions in genes specifying structural and biosynthesis of pilin during cecal colonization by *P. aeruginosa* has been previously demonstrated (Skurnik et al., 2013). Moreover, the surface antigens-like protein ECL_04444 can be qualified as a new virulence factor in *E. cloacae* ATCC 13047 since the corresponding mutant strain was significantly less virulent than the wild type strain in the *G. mellonella* model of infection. This could *a priori* appear contradictory with the Tn-seq results where Tn-insertion in ECL_04444 was positively selected. Nevertheless, it may be likely due to the different experimental procedures leading to the highlighting of colonization or virulence phenotypes. As reported in the literature, the Tn-seq screening is more comparable to competition experiments than to single strain culture assay (Valentino et al., 2014; Verhagen et al., 2014). The host was able to get rid of this mutant more efficiently when it was injected alone than when it was part of a pool of several type of mutant cells. One can then hypothesize that during competitive events, the mutant lacking this surface protein within the population could have fitness advantage by benefiting from the “titration” of the phagocytic cells.

The NAD-dependent epimerase/dehydratase (ECL_02247) enzyme appeared also as an important virulence determinant for *E. cloacae* ATCC 13047. The ECL_02247 gene was one of the most predominant candidates negatively selected after 48 h in *G. mellonella* and the corresponding deletion mutant was less virulent than the parental strain. As systematically reported in Tn-seq data, several metabolic enzymes have been found essential or contributing to the fitness and/or the virulence of bacterial cells (Maroncle et al., 2002; Kamp et al., 2013; Skurnik et al., 2013; Lin et al., 2014; Valentino et al., 2014; Verhagen et al., 2014; Gallagher et al., 2015). Recently, it has been shown that the gene encoding NAD-dependent epimerase/dehydratase, *wcaG*, affects virulence in the Gram negative phytopathogen *Pectobacterium carotovorum* (Islam et al., 2019), but the role of this enzyme in the infection process by *E. cloacae* remains to be elucidate.

Among the mutants for genes contributing to the survival within the host, ECL_00056 (encoding a TetR family transcriptional regulator) deleted strain has reduced capacity to survive in the larvae in co-infection experiments. Our transcriptomic results suggested that this regulator negatively controlled the expression of the downstream operon *eefABC* coding for a tripartite multidrug efflux pump also identified in *Klebsiella* (formerly *Enterobacter*) *aerogenes* (Masi et al., 2005). Moreover, in *K. pneumoniae*, EefABC is involved in gastrointestinal colonization as well as in tolerance response to inorganic acids (Maroncle et al., 2002; Coudeyras et al., 2008). Despite that *eefABC* mutant of *E. cloacae* ATCC 13047 did not revealed neither alteration in MICs of antimicrobial molecules nor modified virulence phenotype, we have shown that *eefABC* expressed in *trans* into the Δ*acrB* hypersusceptible strain of *E. cloacae* partially restored the wild type phenotype (Guérin et al., 2016). Because *eefABC* was over-expressed in the ECL_00056 mutant, it is tempting to speculate that the phenotype observed in coinfection may be due to the deregulation of still uncharacterized ECL_00056-controled-gene(s).

ECL_01421 (encoding a hypothetical protein) was the negative-selected Tn-insertion candidate showing the lowest FC during the infection of larvae of *G. mellonella* (-677) and we revealed that the corresponding mutant was more susceptible to oxidative stress. It is a very short putative ORF (147 bp) and BLAST searches only found homologous sequences in *Enterobacter* species and *Salmonella bongori* genomes (Bao et al., 2014). Nonetheless, it may be possible that ECL_01421 could be an sRNA rather than a coding region or a 3’ untranslated region (UTR) because it is located just 1bp downstream the putative operon *flhBAE* involved in the flagellar synthesis, which appeared particularly important for *in vivo* fitness, as previously discussed.

This first Tn-seq study performed with *E. cloacae* ATCC 13047 led to work out an interesting and powerful tool that allowed us to characterize genes that were important for *in vivo* fitness and virulence of this opportunistic pathogen. The role of these factors should be from now on validated in other clinical isolates of ECC especially from different clusters with different virulence profiles. Analysis of clinical isolates as well as genomic comparisons revealed the occurrence of virulence-associated determinants in *E. cloacae* (Keller et al., 1998; Bingle et al., 2008). The use of our Tn-insertion library could be a pertinent tool to validate these data in different models of infection. Moreover, our results constituted an important resource for further studies on relevant opportunistic traits of this species and laid the foundation for identifying potential antimicrobial targets and antigens with the aim to develop active or passive vaccination.

## Data Availability Statement

This manuscript contains previously unpublished data. The data are available in the NCBI database, BioProject accession number PRJNA627053.

## Author Contributions

FG, VC, and J-CG designed the study, analyzed the data, and wrote the manuscript. BG participated to the TnSeq library construction. FG, CL, CI, MS, MC, and RT performed all other experiments. All authors read and approved the final version of the manuscript.

## Conflict of Interest

The authors declare that the research was conducted in the absence of any commercial or financial relationships that could be construed as a potential conflict of interest.

## Supplementary Material

FIGURE S1 Genetic organization of the selected genes and surrounding regions whose deletion mutants have been created.

FIGURE S2 Growth curves of *E. cloacae* ATCC 13047 and mutant strains at 37°C in LB.

FIGURE S3 **(A)** Percentage of survival after 2 hours at pH 5 at 37°C in LB. No statistical difference was observed compared to the wild type. **(B)** Percentage of autolysis after 10 hours of incubation at 37°C with 0.5% of Triton 100X. No statistical difference was observed compared to the wild type except for the Δ*acrB* mutant used as positive control.

TABLE S1 Primers used in this study.

TABLE S2 MICs (μg/ml) of antibiotics, antiseptics and biocides for *E. cloacae* ATCC 13047 and mutant strains of *E. cloacae*.

TABLE S3 Genes identified as essential for growth in LB.

TABLE S4 Genes identified as essential for the survival within the larvae of *G. mellonella*.

TABLE S5 Genes of *E. cloacae* ATCC 13047 of which Tn mutants have been identified after growth in LB medium and/or after 48h in larvae of *G. mellonella*.

TABLE S6 Tn insertion mutants with a positive phenotype for larvae of *G. mellonella* colonization (changes > +10-fold).

TABLE S7 Tn insertion mutants with a negative phenotype for larvae of *G. mellonella* colonization (changes < −10-fold).

TABLE S8 Tn insertion mutants affected in genes with function is linked with flagella.
