# Supplementary material for: Landscape of in vivo Fitness-Associated Genes of Enterobacter cloacae Complex
Source: Front Microbiol. 2020 Jul 10;11:1609. doi: 10.3389/fmicb.2020.01609 (PMC7365913; doi:10.3389/fmicb.2020.01609)
Supplement: FIGURE S1 — Genetic organization of the selected genes and surrounding regions whose deletion mutants have been created. [file Data_Sheet_1.docx]

**multidrug efflux transport – RND efflux pump**

ECL_00056

TetR family transcriptional regulator

ECL_00054

*eefB*

ECL_00055

*eefA*

ECL_00053

*eefC*

ECL_00094

*two-component system, NarL family*

ECL_00097

putative transcriptional regulator

ECL_00096

Hypothetical protein

ECL_00418

*pilP*

ECL_00095

putative transcriptional

regulatory protein

ECL_00421

Prepilin

ECL_00420

*pilP*

ECL_00419

Type IV pilus

ECL_00413

ECL_00415

*pilM*

ECL_00414

*pilL*

ECL_00416

Type IV pilus

ECL_00417

*pilO*

ECL_01419

*flhA*

ECL_01422

major facilitator transporter

ECL_01420

*flhE*

ECL_01418

*flhB*

ECL_01421

Hypothetical protein

cytochrome bd-I oxidase

ECL_02048

*cydB*

ECL_02047

*cydA*

ECL_02043 - 02045

Hypothetical proteins

ECL_02046

Putative catalase

ECL_02245

Hypothetical protein

ECL_02248

*lysR*

transcriptional regulator

ECL_02247

NAD-dependent epimerase/ dehydratase

ECL_02246

Hypothetical protein

ECL_03232

*fliR*

ECL_03227 / 28 /29 30

*fliM fliN fliO fliP*

ECL_03225

*fliK*

ECL_03224

*fliJ*

ECL_03226

*fliL*

ECL_03223

*fliI*

ECL_03219

*fliE*

ECL_03220

*fliF*

ECL_03222

*fliH*

ECL_03221

*fliG*

ECL_04444

Opacity protein and surface antigens-like protein

ECL_04445

outer membrane usher protein

ECL_04443

phage integrase family site-specific recombinase

**FIGURE S1** Genetic organization of the selected genes and surrounding regions whose deletion mutants have been created.
