# Supplementary figures and images for: Landscape of in vivo Fitness-Associated Genes of Enterobacter cloacae Complex
Source: Front Microbiol. 2020 Jul 10;11:1609. doi: 10.3389/fmicb.2020.01609 (PMC7365913; doi:10.3389/fmicb.2020.01609)

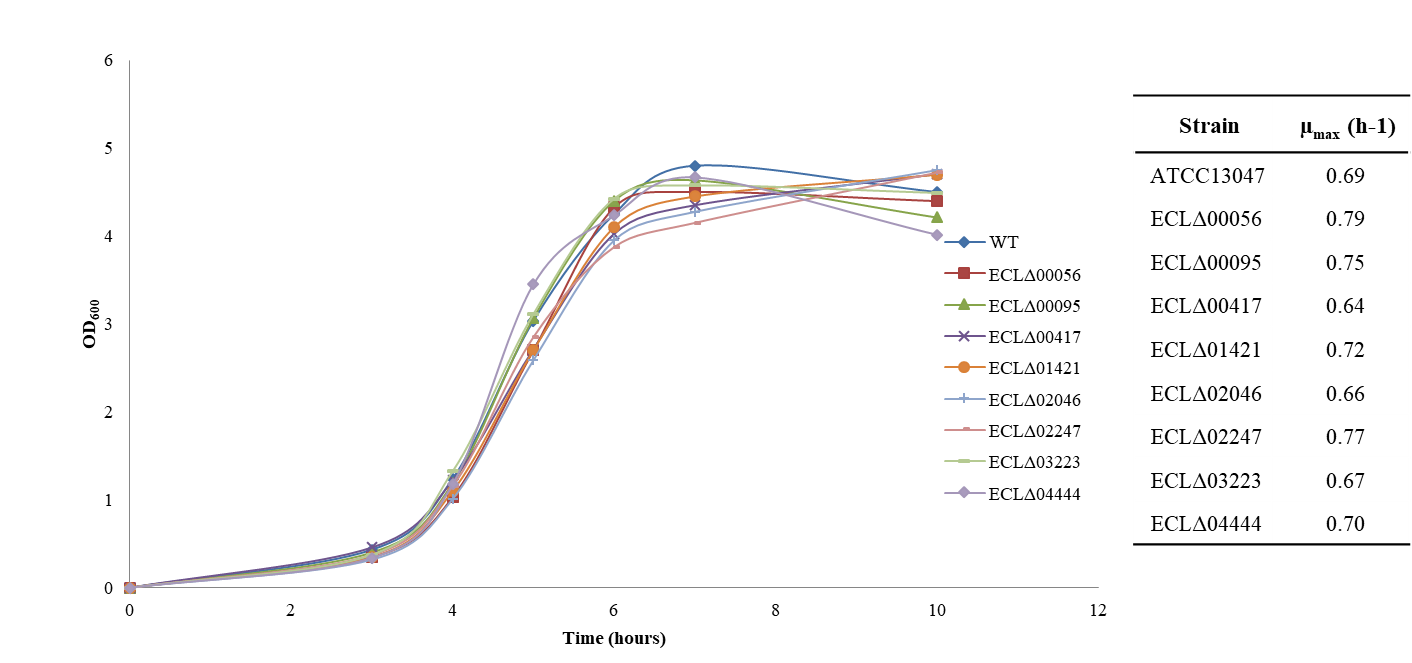


**FIGURE S2** Growth curves of *E. cloacae* ATCC 13047 and mutant strains at 37°C in LB.

Supplement: FIGURE S2 — Growth curves of E. cloacae ATCC 13047 and mutant strains at 37°C in LB. [file Data_Sheet_2.docx]
