# Supplementary material for: Landscape of in vivo Fitness-Associated Genes of Enterobacter cloacae Complex
Source: Front Microbiol. 2020 Jul 10;11:1609. doi: 10.3389/fmicb.2020.01609 (PMC7365913; doi:10.3389/fmicb.2020.01609)
Supplement: FIGURE S3 — (A) Percentage of survival after 2 hours at pH 5 at 37°C in LB. No statistical difference was observed compared to the wild type. (B) Percentage of autolysis after 10 hours of incubation at 37°C with 0.5% of Triton 100X. No statistical difference was observed compared to the wild type except for the ΔacrB mutant used as positive control. [file Data_Sheet_3.docx]

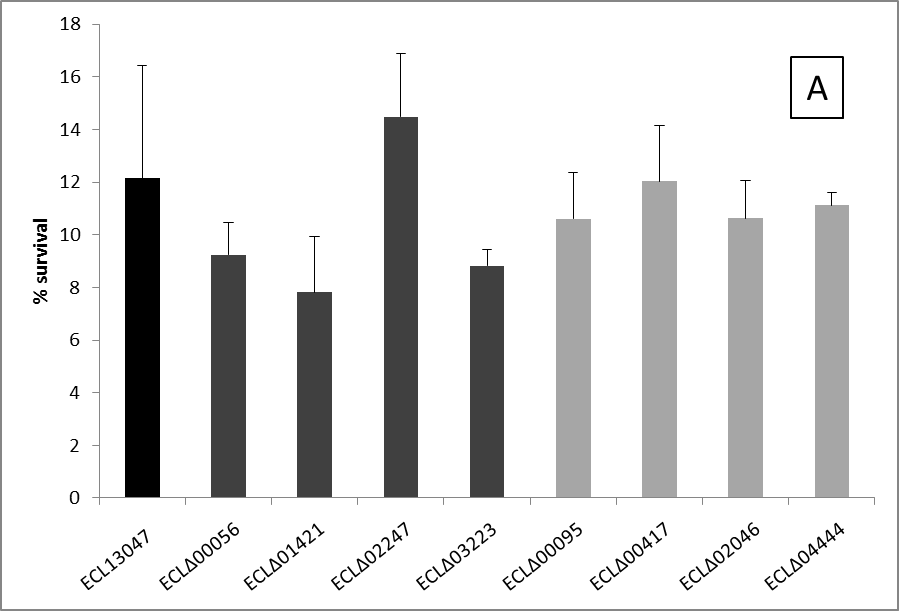


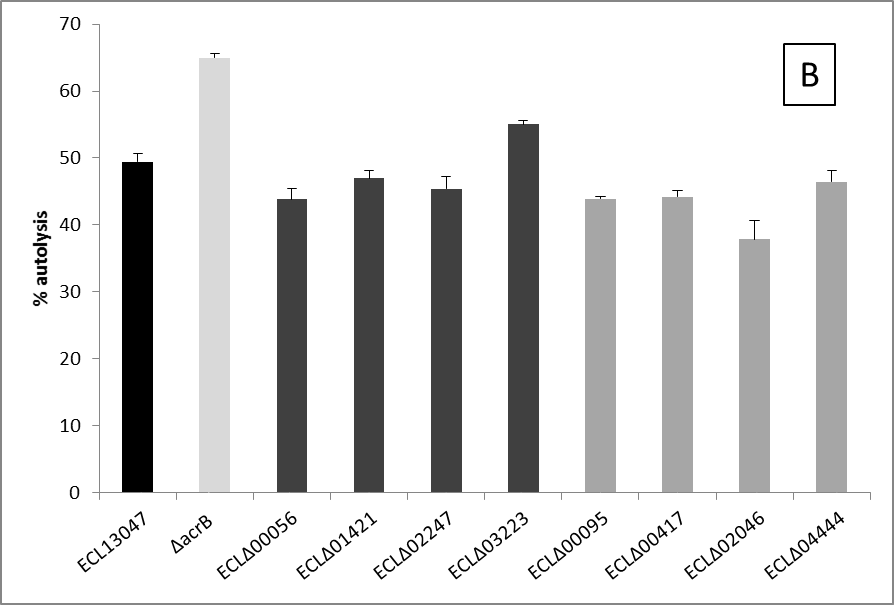


**FIGURE S3** A: percentage of survival after 2 hours at pH 5 at 37¨C in LB. No statistical difference was observed compared to the wild type. B: percentage of autolysis after 10 hours of incubation at 37°C with 0.5% of Triton 100X. No statistical difference was observed compared to the wild type except for the ∆*acrB* mutant used as positive control.
