# Supplementary material for: Landscape of in vivo Fitness-Associated Genes of Enterobacter cloacae Complex
Source: Front Microbiol. 2020 Jul 10;11:1609. doi: 10.3389/fmicb.2020.01609 (PMC7365913; doi:10.3389/fmicb.2020.01609)
Supplement: TABLE S1 — Primers used in this study. [file Table_1.docx]

**TABLE S1**: Primers used in this study

| Primers | Sequence (5’- 3’) | Use |  |
| --- | --- | --- | --- |
| ECL-pKD4-00056_F | CAAAACGTATAATGAGAACGATCATTCTCACTTTTACCCGGTAATCGACGgtgtaggctggagctgcttc | ECL_00056 deletion |  |
| ECL-pKD4-00056_R | CCAGAAATGGTAAACCGGGAAATTACCTGTTCCCGTTAATTATTCACATGCGAcatatgaatatcctccttag |  |  |
| ECL-00056v_F | TTCACCTTTTCGTACGCCGA | Verification of ECL_00056 deletion |  |
| ECL-00056v_R | CAGTTCACTGACGACGGACA |  |  |
| ECL-pKD4-00095_F | CGGTCACATTAATCCTTAAACCGGCGTTTACAGGAGGTATTTAACGAGTCTTgtgtaggctggagctgcttc | ECL_00095 deletion |  |
| ECL-pKD4-00095_R | ATGATCCTGAATAAAGTAATCTGACATACAACGGTTTGGTTCCGTCATTGCcatatgaatatcctccttag |  |  |
| ECL-00095v_F | CGTGGGGAAAGGTAGCATGA | Verification of ECL_00095 deletion |  |
| ECL-00095v_R | ACGGTTCGTACTTCTCCAGC |  |  |
| ECL-pKD4-00417_F | GGGCCTCGCCGGACACCCACTGCGTGACGGCCGGCAGAGGCAGCGCTTCCgtgtaggctggagctgcttc | ECL_00417 deletion |  |
| ECL-pKD4-00417_R | GCTCCCCTTCATGGTGTATCCGAACTGCCCCTGCGGGCTCATGGTGATGGcatatgaatatcctccttag |  |  |
| ECL-00417v_F | CAGAACGTCTCGGCGGATAA | Verification of ECL_00417 deletion |  |
| ECL-00417v_R | GCAGGGATGTCGCCAGATAAG |  |  |
| ECL-pKD4-01421_F | GCAGAGTAATTCCGTTATCGTTAACTACCGCTAGTTGCCTGCCTCAACGCCCgtgtaggctggagctgcttc | ECL_01421 deletion |  |
| ECL-pKD4-01421_R | TACACGGCTACAGTAGAATTAACGCACTGTGGAAACCTTTCTGTTTCTGCCAcatatgaatatcctccttag |  |  |
| ECL-01421v_F | ACTACCCAGGCGTTTAACGG | Verification of ECL_01421 deletion |  |
| ECL-01421v_R | AAGGTCGCTTCAATCGGTGT |  |  |
| ECL-pKD4-02046_F | CACAGGAGAGTGTATGTTTCGACACATCAAACAGCTGCAATACACGGTTCGgtgtaggctggagctgcttc | ECL_02046 deletion |  |
| ECL-pKD4-02046_R | ACGATGGGGCACGAGAATTACCAGGCAGCGGGAGCTGCCTGGCAAAACCGGcatatgaatatcctccttag |  |  |
| ECL-02046v_F | AATTGCCTGCTACACCTCCC | Verification of ECL_02046 deletion |  |
| ECL-02046v_R | AAACTGAGAGAACCCGCTCC |  |  |
| ECL-pKD4-02247_F | GATCACACTGTGTCTGTCACCCCTTTCGGAGAAACACCATGAATACGACGgtgtaggctggagctgcttc | ECL_02247 deletion |  |
| ECL-pKD4-02247_R | ACAGTCATGAGCGAGTATAACGGGTTCGTTCCTGAGAACAACATCCCCCTGcatatgaatatcctccttag |  |  |
| ECL-02247v_F | CTTCATCCTGCGCGTTGTAC | Verification of ECL_02247 deletion |  |
| ECL-02247v_R | CGTTCGGGTAAAACTGCACC |  |  |
| ECL-pKD4-03223_F | CCGCACGCCTCACTCGCTGGCTCAACACGCTCGATAACTTTGAAACGAAGATGgtgtaggctggagctgcttc | ECL_03223 deletion |  |
| ECL-pKD4-03223_R | GACCGTCGCCCTCCACCTGTGTTACACCTGCGGGAAAATCAGCTCCAGAGCcatatgaatatcctccttag |  |  |
| ECL-03223v_F | GAAGAGAGTCTCGGTGCCAC | Verification of ECL_03223 deletion |  |
| ECL-03223v_R | GCCTTCTCCAGCGTCTGAAT |  |  |
| ECL-pKD4-04444_F | TCGGGACTGCTTATGATTCGTCTCACTGACTGTTTTTCAATGGAGTGTAGgtgtaggctggagctgcttc | ECL_04444 deletion |  |
| ECL-pKD4-04444_R | CCGCACTCCCACGGTGTTGGCCCTGTTTTAAAGCAGTGACGGCCACAACAGGcatatgaatatcctccttag |  |  |
| ECL-04444v_F | TGAACCTCAGTATGGTGCGC | Verification of ECL_04444 deletion |  |
| ECL-04444v_R | CAGGAGCGCAATAAAACGGG |  |  |
| LIB_AdaptT | TTCCCTACACGACGCTCTTCCGATCTNN | Construction of DNA library |  |
| LIB_AdaptB | AGATCGGAAGAGCGTCGTGTAGGGAA |  |  |
| LIB_PCR_5 | CAAGCAGAAGACGGCATACGAAGACCGGGGACTTATCATCCAACCTGT |  |  |
| LIB_PCR_3 | AATGATACGGCGACCACCGAACACTCTTTCCCTACACGACGCTCTTCCGATCT |  |  |
| LIB_AdaptT_1 | TTCCCTACACGACGCTCTTCCGATCTAAAGGNN |  |  |
| LIB_AdaptB_1 | CCTTTAGATCGGAAGAGCGTCGTGTAGGGAA |  |  |
| LIB_AdaptT_2 | TTCCCTACACGACGCTCTTCCGATCTTTTCCNN |  |  |
| LIB_AdaptB_2 | GGAAAAGATCGGAAGAGCGTCGTGTAGGGAA |  |  |
| LIB_AdaptT_3 | TTCCCTACACGACGCTCTTCCGATCTGGGAANN |  |  |
| LIB_AdaptB_3 | TTCCCAGATCGGAAGAGCGTCGTGTAGGGAA |  |  |
| LIB_AdaptT_4 | TTCCCTACACGACGCTCTTCCGATCTCCCTTNN |  |  |
| LIB_AdaptB_4 | AAGGGAGATCGGAAGAGCGTCGTGTAGGGAA |  |  |
| LIB_AdaptT_5 | TTCCCTACACGACGCTCTTCCGATCTCTATCNN |  |  |
| LIB_AdaptB_5 | GATAGAGATCGGAAGAGCGTCGTGTAGGGAA |  |  |
|  |  |  |  |

F:Forward,R:Reverse
