# Supplementary material for: Landscape of in vivo Fitness-Associated Genes of Enterobacter cloacae Complex
Source: Front Microbiol. 2020 Jul 10;11:1609. doi: 10.3389/fmicb.2020.01609 (PMC7365913; doi:10.3389/fmicb.2020.01609)
Supplement: TABLE S2 — MICs (μg/ml) of antibiotics, antiseptics and biocides for E. cloacae ATCC 13047 and mutant strains of E. cloacae. [file Table_2.docx]

**TABLE S2** MICs (µg/ml) of antibiotics, antiseptics and biocides for *E. cloacae* ATCC 13047 and mutant strains.

|  | ECL13047 | ECL∆00056 | ECL∆00095 | ECL∆00417 | ECL∆01421 | ECL∆02046 | ECL∆02247 | ECL∆03149 | ECL∆04444 |
| --- | --- | --- | --- | --- | --- | --- | --- | --- | --- |
| **Antibiotics** |  |  |  |  |  |  |  |  |  |
| Amoxicillin | >256 | >256 | >256 | >256 | >256 | >256 | >256 | >256 | >256 |
| Piperacillin | 4 | 4 | 4 | 4 | 4 | 4 | 4 | 4 | 4 |
| Cefoxitin | 256 | 256 | 256 | 256 | 256 | 256 | 256 | 256 | 256 |
| Cefotaxime | 2 | 2 | 2 | 2 | 2 | 2 | 2 | 2 | 2 |
| Ceftriaxone | 2 | 2 | 2 | 2 | 2 | 1 | 1 | 1 | 1 |
| Cefepime | 0,01 | 0,01 | 0,01 | 0,01 | 0,01 | 0,01 | 0,01 | 0,01 | 0,01 |
| Aztreonam | 1 | 1 | 1 | 1 | 1 | 1 | 1 | 1 | 1 |
| Imipenem | 0,12 | 0,12 | 0,12 | 0,12 | 0,12 | 0,12 | 0,12 | 0,12 | 0,12 |
| Ertapenem | 0,03 | 0,03 | 0,03 | 0,03 | 0,03 | 0,03 | 0,03 | 0,03 | 0,03 |
| Gentamicin | 0,12 | 0,12 | 0,12 | 0,12 | 0,25 | 0,12 | 0,12 | 0,12 | 0,12 |
| Tobramycin | 0,5 | 0,5 | 0,5 | 0,5 | 0,5 | 0,5 | 0,5 | 0,5 | 0,5 |
| Amikacin | 1 | 1 | 1 | 1 | 1 | 1 | 1 | 1 | 1 |
| Norfloxacin | 0,12 | 0,12 | 0,12 | 0,12 | 0,12 | 0,12 | 0,12 | 0,12 | 0,12 |
| Levofloxacin | 0,06 | 0,03 | 0,03 | 0,03 | 0,03 | 0,03 | 0,03 | 0,03 | 0,03 |
| Ciprofloxacin | 0,03 | 0,03 | 0,03 | 0,03 | 0,03 | 0,03 | 0,03 | 0,03 | 0,03 |
| Moxifloxacin | 0,06 | 0,06 | 0,06 | 0,06 | 0,06 | 0,06 | 0,06 | 0,06 | 0,06 |
| Tetracycline | 2 | 2 | 2 | 2 | 2 | 2 | 2 | 2 | 2 |
| Tigecycline | 0,5 | 0,5 | 0,5 | 0,5 | 0,5 | 0,5 | 0,5 | 0,5 | 0,5 |
| Cotrimoxazole | 2 | 2 | 2 | 2 | 2 | 2 | 2 | 2 | 2 |
| Chloramphenicol | 8 | 8 | 8 | 8 | 8 | 8 | 8 | 8 | 8 |
| Fusidic acid | 256 | 256 | 256 | 256 | 256 | 256 | 256 | 256 | 256 |
| Nitrofurantoin | 32 | 32 | 32 | 32 | 32 | 32 | 32 | 32 | 32 |
| Erythromycin | 256 | 256 | 256 | 256 | 256 | 256 | 256 | 256 | 256 |
| **Antiseptics** |  |  |  |  |  |  |  |  |  |
| Benzalkonium chloride | 32 | 32 | 32 | 32 | 32 | 32 | 32 | 32 | 32 |
| Cetyltrimethylammunium bromide (CTAB) | 16 | 16 | 16 | 16 | 16 | 32 | 32 | 32 | 32 |
| Chlorhexidine | 2 | 2 | 2 | 2 | 2 | 2 | 2 | 2 | 2 |
| Tetraphenylphosphosnium | >1024 | >1024 | >1024 | >1024 | >1024 | >1024 | >1024 | >1024 | >1024 |
| **Biocides** |  |  |  |  |  |  |  |  |  |
| Acridin orange | 512 | 512 | 512 | 512 | 512 | 512 | 512 | 512 | 512 |
| Acriflavin | 64 | 64 | 64 | 64 | 64 | 64 | 64 | 64 | 64 |
| Violet Cristal | 32 | 32 | 32 | 32 | 32 | 32 | 32 | 32 | 32 |
| Ethidium bromide | 1024 | 1024 | 1024 | 1024 | 1024 | 1024 | 1024 | 1024 | 1024 |
| Rhodamin | >1024 | >1024 | >1024 | >1024 | >1024 | >1024 | >1024 | >1024 | >1024 |
| **Metal** |  |  |  |  |  |  |  |  |  |
| Copper | 1024 | 1024 | 1024 | 1024 | 1024 | 1024 | 1024 | 1024 | 1024 |
| Zinc | 128 | 128 | 128 | 128 | 128 | 128 | 128 | 128 | 128 |
| Manganese | >1024 | >1024 | >1024 | >1024 | >1024 | >1024 | >1024 | >1024 | >1024 |
| Silver | 2 | 2 | 2 | 2 | 1 | 1 | 1 | 1 | 1 |
| Mercury | 4 | 4 | 4 | 4 | 4 | 4 | 4 | 4 | 8 |
| **Other** |  |  |  |  |  |  |  |  |  |
| Sodium dodecyl sulfate (SDS) | 1 | 1 | 1 | 1 | 1 | 1 | 1 | 1 | 1 |
